# Efficacy and safety of *Saccharomyces boulardii* as an adjuvant therapy for the eradication of *Helicobacter pylori*: a meta-analysis

**DOI:** 10.3389/fcimb.2025.1441185

**Published:** 2025-02-12

**Authors:** Manning Li, Ying Xie

**Affiliations:** Department of Gastroenterology, Shengjing Hospital of China Medical University, Shenyang, Liaoning, China

**Keywords:** *Saccharomyces boulardii*, *Helicobacter pylori*, meta-analysis, eradication rate, adverse effects

## Abstract

**Background:**

*Helicobacter pylori* (*H. pylori*) is highly prevalent worldwide and is closely associated with many gastric conditions. Current methods for eradicating *H. pylori* include triple or quadruple therapy, including antibiotics, proton pump inhibitors, and bismuth agents; however, with antibiotic abuse and increased drug resistance rates, the effectiveness of traditional methods is gradually decreasing, with many adverse effects such as abdominal pain, diarrhea, and intolerance. In recent years, there has been controversy regarding whether adding *Saccharomyces boulardii* (*S. boulardii*) to traditional therapies is beneficial for eradicating *H. pylori*.

**Aim:**

To evaluate the efficacy and safety of *S. boulardii* as an adjuvant therapy for the eradication of *H. pylori*.

**Methods:**

We systematically searched the PubMed and Web of Science databases from January 2002 to January 2023. The primary outcome was the *H. pylori* eradication rate. The secondary outcomes included total adverse effects, abdominal pain, diarrhea, bloating, constipation, nausea, vomiting, taste disorders, and other adverse reactions. We evaluated the included studies for publication bias and heterogeneity. Fixed- and random-effects models were used for studies without and with heterogeneity, respectively, to calculate the risk ratios (RRs) and conduct sensitivity and subgroup analyses.

**Results:**

Nineteen studies comprising 5,036 cases of *H. pylori* infection were included in this meta-analysis. The addition of *S. boulardii* to traditional therapy significantly improved the *H. pylori* eradication rate [RR=1.11, 95% confidence interval (CI): 1.08–1.15] and reduced the incidence of total adverse effects (RR=0.49, 95% CI: 0.37–0.66), diarrhea (RR=0.36, 95% CI: 0.26–0.48), abdominal distension (RR=0.49, 95% CI: 0.33–0.72), constipation (RR=0.38, 95% CI: 0.26–0.57), and nausea (RR=0.50, 95% CI: 0.37–0.68). However, it did not reduce the occurrence of abdominal pain, vomiting, or taste disorders.

**Conclusions:**

*S. boulardii* supplementation in traditional eradication therapy significantly improves the *H. pylori* eradication rate and reduces the total adverse effects and incidence of diarrhea, bloating, constipation, and nausea.

**Systematic review registration:**

Prospero, identifier CRD42024549780.

## Introduction

*H. pylori* is a Gram-negative bacterium that is closely associated with chronic gastritis, peptic ulcers, gastric cancer, and gastric mucosal tissue-related lymphoma. It is classified as a Class I carcinogen by the World Health Organization (Peek, 2013). Approximately 50% of people worldwide are infected with *H. pylori* (Mesquita et al., 2022; Katelaris et al., 2023), and the infection and recurrence rates in developing countries are higher than those in developed countries (Sun and Zhang, 2019). In China, the total prevalence rate is 44.2%, with an estimated 589 million people infected with *H. pylori* (Ren et al., 2022).Currently, the mainstream method for eradicating *H. pylori* is triple or quadruple therapy, including antibiotics, proton pump inhibitors, and bismuth agents (Helicobacter pylori Study Group, C. S. o. G and Chinese Medical Association, 2022). The triple therapy proposed at the first Maastricht Conference, including PPI, clarithromycin and amoxicillin, is currently the most common therapy (Malfertheiner et al., 2012).However, with the abuse of antibiotics and the increase in drug resistance rates, the effectiveness of this traditional method is gradually decreasing, with many adverse effects such as abdominal pain, diarrhea, aggravation of nausea or vomiting and intolerance (Flores-Treviño et al., 2018).The consensus states that if the resistance rate of clarithromycin in a region is greater than 15%, the triple therapy of PPI, clarithromycin and amoxicillin should be abandoned and replaced with PPI, metronidazole and amoxicillin, or a quadruple therapy containing bismuth: bismuth, PPI, tetracycline and metronidazole. The consensus also mentioned that *S. boulardii* has a certain effect on improving *H. pylori* infection.

*S. boulardii* is a yeast that has been isolated from the peel of tropical fruits and is commonly used to treat gastrointestinal diseases such as diarrhea (Kaźmierczak-Siedlecka et al., 2020; Pais et al., 2020). In recent years, domestic and international studies have shown that *S. boulardii* has a positive effect on the eradication of *H. pylori* (Szajewska et al., 2015; Seddik et al., 2019; Yang et al., 2022). In 2017, Yang et al. found that *S. boulardii* could inhibit the formation of gastric lymphatic follicles caused by *H. pylori* infection, thereby reducing the incidence of adverse effects (Lazo-Vélez et al., 2018). Previous studies have also indicated that *S. boulardii* can directly destroy *H. pylori* cells, resulting in cell destruction and damage. It produces substances, such as short chain fatty acids, that inhibit *H. pylori* growth (McFarland, 2010).Moreover, although *S. boulardii* cannot completely eradicate *H. pylori*, it can reduce the colonization of *H. pylori* in the intestines of children (Namkin et al., 2016). The possible mechanism for this is *S. boulardii*’s expression of neuraminidase activity selective for α2,3-linked sialic acid, which can inhibit the adhesion of *H. pylori* to duodenal epithelial cells (Sakarya and Gunay, 2014). Also *S. boulardii* has been shown to stabilize the tight junction of gastric mucosal epithelial cells, stimulating SIgA response and strengthening the gastric mucosal barrier (Buts et al., 1990; Dahan et al., 2003).Moreover, some theories suggest that *S. boulardii* can reduce the abundance of antibiotic-resistance genes and reduce the unsatisfactory traditional treatment effects caused by antibiotic resistance (Cifuentes et al., 2022). To further clarify the role of *S. boulardii* in the eradication of *H. pylori*, we conducted a retrospective meta-analysis of randomized controlled trials published between January 2002 and January 2023 and evaluated the impact of adding *S. boulardii* to traditional methods for the eradication of *H. pylori* infection.

## Materials and methods

### Data sources and literature search

We systematically searched the PubMed and Web of Science databases for literature published from January 2002 to January 2023, using the search terms “*H. pylori*” and “*S. boulardii*.” In total, 125 articles were detected.

### Study selection criteria

The inclusion criteria were as follows: (1) randomized controlled trials; (2) *H. pylori* infection patients positive for *H. pylori* antigens or with a positive 14C breath test; (3) a control group consisting of standard triple therapy, quadruple therapy, sequential therapy, or the addition of a placebo to the standard therapy; (4) experimental group treated with the addition of *S. boulardii* to the standard therapy; and (5) outcomes including the eradication rate of *H. pylori*, overall adverse effects, abdominal pain, diarrhea, bloating, nausea and vomiting. The exclusion criteria were as follows: (1) therapy consisting of a combination of *S. boulardii* and other non-*S. boulardii* probiotics; (2) animal experiments; (3) meta-analyses, reviews, and conference abstracts; (4) repetitive literature published in different publications; (5) non-randomized controlled trials; and (6) inability to obtain full text or incomplete data. Two reviewers independently screened the studies by reading the titles and abstracts and obtained the full texts of the relevant articles. Any differences were resolved by consensus.

### Data extraction

The following data were extracted from each eligible study: first author, year of publication, study location, patient age, number of participants, eradication protocol, *S. boulardii* protocol, and follow-up duration. Two reviewers independently extracted data from each selected study and any differences were resolved by consensus.

### Evidence quality and bias test

**Figure 1:**
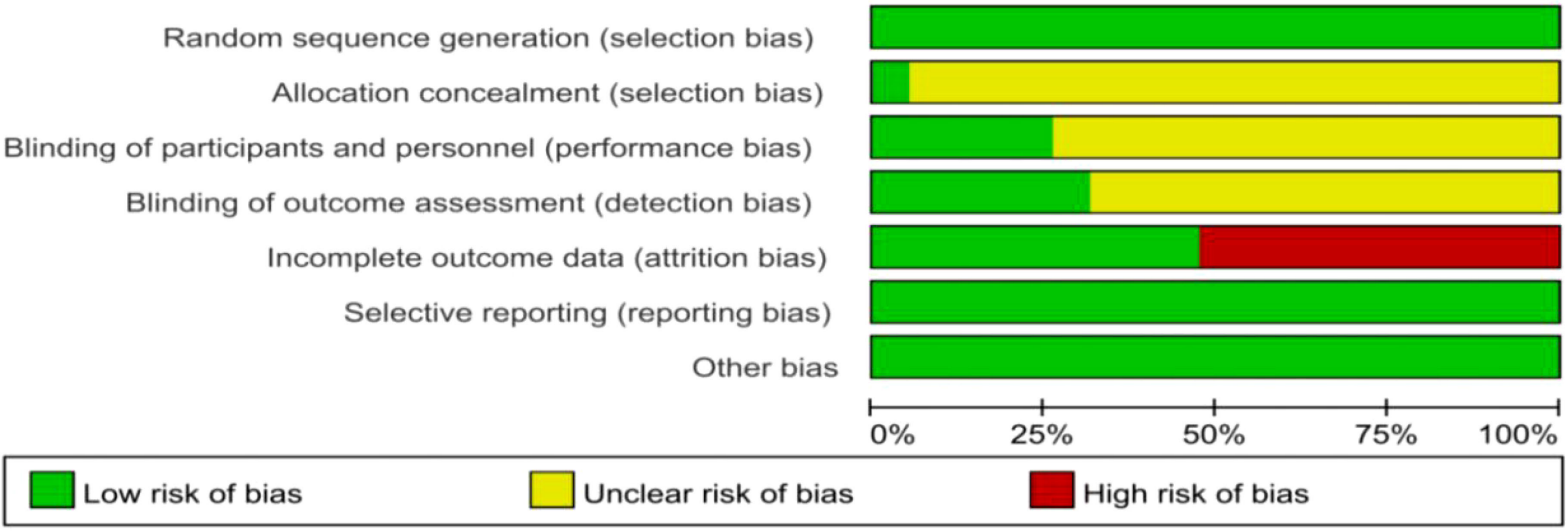
Cochrane Quality Evaluation Form. Studies were analyzed for a variety of bias using the tools in Review Manager software (Version 5.4).

A Cochrane quality evaluation form (**Figure 1**) was developed to evaluate the quality of the studies. Using the Egger test, we found that the selected studies had no publication bias, indicating that the conclusions of this study were accurate and reliable.

### Meta-analysis and statistical analysis

All statistical analyses were conducted using Review Manager software (version 5.4; Cochrane Collaboration, Oxford, UK), and the results were expressed as the risk ratio (RR) with a 95% confidence interval (CI). Statistical significance was set at P<0.05.

We used I^2^ statistics to evaluate heterogeneity, with I^2^ values of 0%–25%, 26%–50%, 51%–75%, and >75% indicating no heterogeneity, low heterogeneity, medium heterogeneity, and high heterogeneity, respectively. For studies without heterogeneity, a fixed-effects model was used to calculate the RRs. In contrast, for studies with heterogeneity, a random-effects model was used to calculate the RRs, and sensitivity and subgroup analyses were performed.

## Results

### Literature search process and results

**Figure 2:**
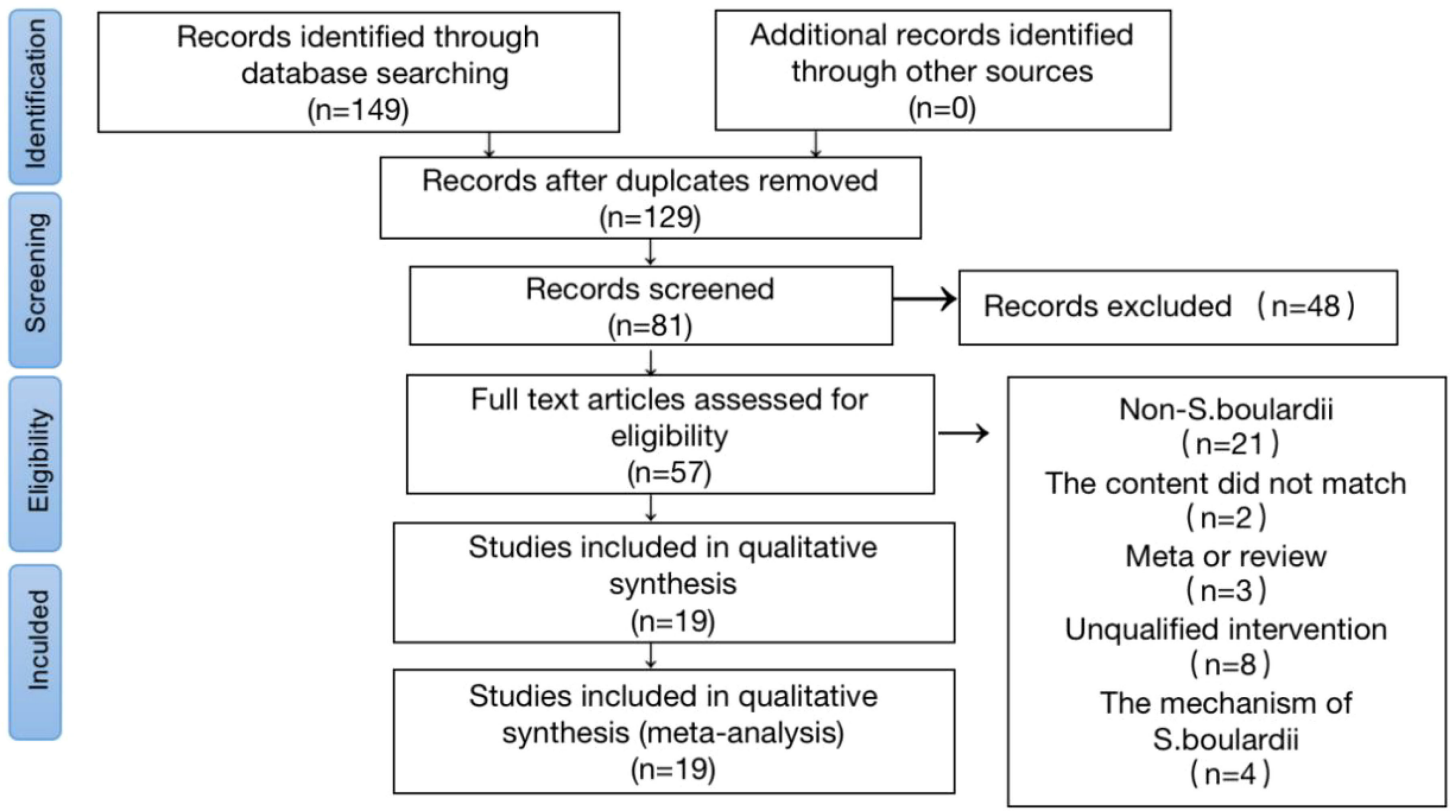
Flow Chart of Literature Screening. A flow diagram of articles retrieved and inclusion progress through the stage of meta-analysis.

A total of 149 studies published between January 2002 and January 2023 were included. After removing duplicate and unrelated records, 57 full-text articles were evaluated, of which 38 studies were excluded based on the selection criteria. Therefore, the final meta-analysis included 19 studies (Cremonini et al., 2002; Duman et al., 2005; Cindoruk et al., 2007; Hurduc et al., 2009; Song et al., 2010; Zojaji et al., 2013; Zhao et al., 2014; Bin et al., 2015; Szajewska et al., 2015; Wang and Yu, 2015; Chotivitayatarakorn et al., 2017; Zhu et al., 2017; Zhu et al., 2018; He et al., 2019; He et al., 2019; Seddik et al., 2019; Chang et al., 2020; Zhao et al., 2021; Naghibzadeh et al., 2022). A PRISMA flowchart of the study selection process is shown in **Figure 2**.

### Characteristics of the included studies

A total of 19 randomized controlled trials involving 5,036 patients (**Table 1**) were included, of which 9 were domestic studies and 10 were foreign studies; 15 were adult studies and 4 were child studies; and 13 used triple therapy, 5 used quadruple therapy, and 1 used sequential therapy. Moreover, regarding the duration of therapy, four studies used 7-day therapy, 13 studies used 14-day therapy, one study collected data on both 7-day and 14-day therapy, and one study used 10-day therapy.

**Table 1:** Basic characteristics of included study.

First author and year	Country	Publication	No. of patients	Patients	Eradication regimen	*S. boulardii* regimen (dose, duration)	Follow‐up post‐treatment time
Yang, G. B. 2022	China	Full paper	497	Adults	Rabeprazole 20mg bid,amoxicillin 1.0g bid,clarithromycin 0.5g bid,10d	0.5g bid,14d	14d,44d
Naghibzadeh, N. 2022	Iran	Full paper	156	Adults	Amoxicillin 1.0g bid,clarithromycin 0.5g bid,PPI 40mg bid,bismuth subcitrate 240mg bid,14d,PPI 40mg bid for another 14d	0.25g bid,14d	7d,14d,21d,28d
Zhao, Yuchong 2021	China	Full paper	360	Adults	Esomeprazole 20mg bid,amoxicillin 1.0g bid,clarithromycin 0.5g bid,bismuth potassium citrate 600mg bid,14d	0.5g bid,14d	4w-12w
Chang, Y. W. 2020	Korea	Full paper	183	Adults	Pantoprazole 40mg bid,amoxicillin 1.0g bid,clarithromycin 0.5g bid,7d	0.25g tid,4w	4w
Seddik, H. 2019	Morocco	Full paper	199	Adults	Omeprazole 20mg bid, amoxicillin 1.0g bid for the first 5d, followed by omeprazole 20mg bid, clarithromycin 0.5g bid,metronidazole 0.5g bid for the remaining 5d	0.25g bid,10d	4w
He, Jin'e 2019	China	Full paper	120	Children	Omeprazole 0.7~0.8mg/(kg.d),amoxicillin 50mg/(kg.d),clindamycin 20mg/(kg.d),14d	0.25g bid,14d	4w
He, C. X. 2019	China	Full paper	300	Adults	Pantoprazole 40mg bid,amoxicillin 1.0g bid ,furazolidone 100mg bid,bismuth potassium citrate 220mg bid,14d	0.5g bid,14d	4w
Zhu, X. Y. 2018	China	Full paper	360	Adults	Bismuth potassium citrate 220mg bid, esomeprazole 10mg bid, amoxicillin 1.0g bid, furazolidone 100mg bid, 10 d	0.5g bid,14d	4w
Zhu, X. Y. 2017	China	Full paper	240	Adults	Bismuth potassium citrate 220mg bid, rabeprazole 10mg bid, amoxicillin 1.0g bid, furazolidone 100mg bid, 10 d	0.5g bid,14 d,28 d	4w
Chotivitayatarakorn, Peranart 2017	Thailand	Full paper	108	Adults	Dexlansoprazole 60mg bid, moxifloxacin 400mg qd, clarithromycin 1.0g qd, 7d,14 d	282.5mg bid,7d,14d	4w
Wang, Likun 2015	China	Full paper	240	Adults	Pantoprazole 40mg bid,amoxicillin 1.0g bid ,clarithromycin 0.5g bid,14d	282.5mg bid,14d,21d,28d	1w,2w,3w,4w
Bin, Zhang 2015	China	Full paper	194	Children	Omeprazole (NR) , clarithromycin (NR) , amoxicillin (or metronidazole; NR) , 14 d	0.25g bid,14d	4w
Zhao, Hong-Mei 2014	China	Full paper	240	Children	Omeprazole 0.7-0.8 mg/(kg.d) qd, amoxicillin 40 mg/(kg.d) tid, clarithromycin 15 mg/(kg.d) bid, 14 d	0.25g bid,14d	4w
Zojaji, Homayoun 2013	Iran	Full paper	160	Adults	Omeprazole 20mg bid,amoxicillin 1.0g bid, clarithromycin 500mg bid, 14 d	0.25g bid,14d	8w
Song, M. J. 2010	Korea	Full paper	991	Adults	Omeprazole 20mg bid, amoxicillin 1.0g bid, clarithromycin 500mg bid, 7 d	0.25g tid,28d	4w
Hurduc, V. 2009	Romania	Full paper	90	Children	Omeprozole or esomeprazole 0.5 mg/kg bid, 21d, clarothromycin 7.5 mg/kg bid, amoxicillin 25 mg/kg bid, 7‐10 d	0.25g bid,28d	4-6w
Cindoruk, M. 2007	Turkey	Full paper	124	Adults	Lansoprazole 30mg bid, amoxicillin 1.0g bid, clarithromycin 500mg bid, 14 d	0.5g bid,14d	6w
Duman, D. G. 2005	Turkey	Full paper	389	Adults	Omeprazole 20mg bid, amoxicillin 1.0g bid, clarithromycin 500mg bid, placebo 250mg bid,14 d	0.5g bid,14d	15-45d
Cremonini, F. 2002	Italy	Full paper	85	Adults	Rabeprazole 20mg bid, clarithromycin 500mg bid, tinidazole 500mg bid, placebo 2 sachets/day, 7 d	5x10^9^/CFU bid, 14d	5-7w

bid, twice daily; qd, once daily; d,day; w, week; CFU, colony‐forming units; NR, not reported; PPI, Proton Pump Inhibitors.

### Primary outcome: *H. pylori* eradication rate

#### Eradication rate

**Figure 3:**
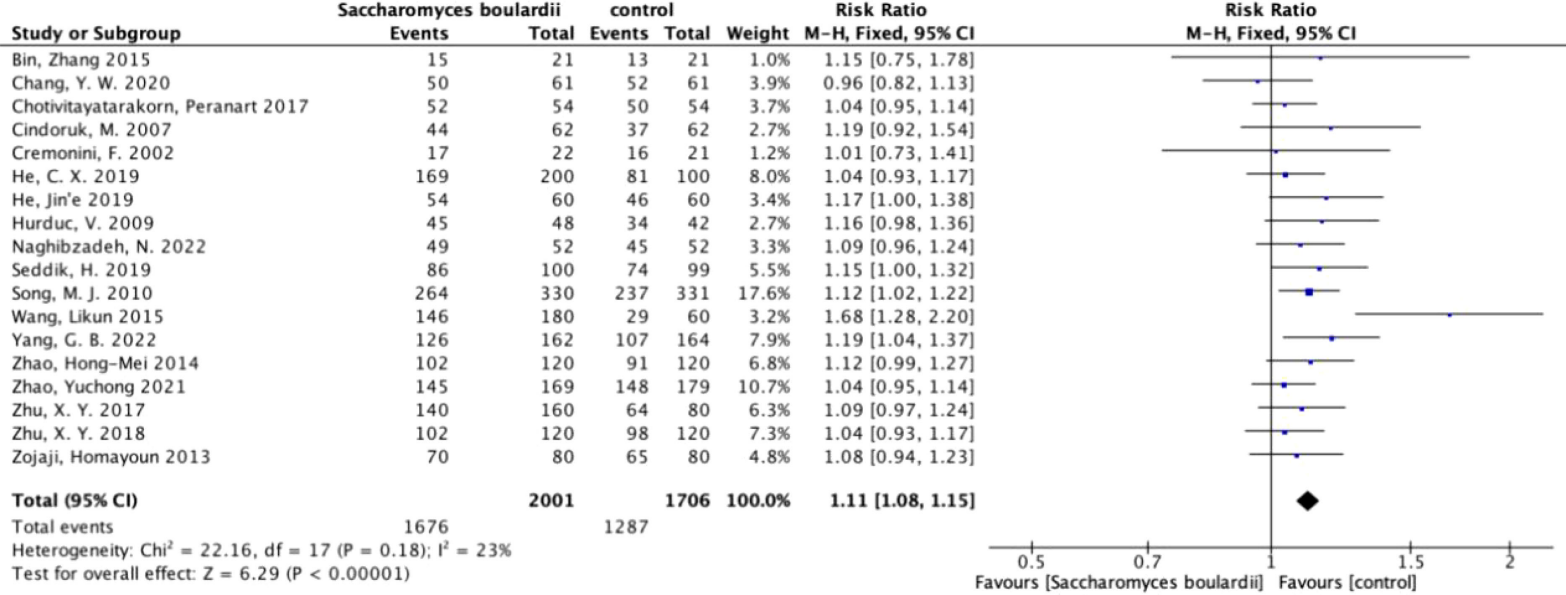
Forest plot of eradication rate. Forest plot analyzing the eradication rate of *S. boulardii* as an adjuvant therapy for *H. pylori*.

Among the 19 included articles, 18 statistically analyzed the eradication rate of *H. pylori*. We performed ITT analysis on 18 articles (**Figure 3**) and the results showed no heterogeneity (I^2^ = 23%, P=0.18). Using a fixed-effects model, we found that the eradication rate was increased by 11% when using *S. boulardii* supplementation (RR=1.11, 95% Cl: 1.08–1.15, P<0.0001), with no observed bias (Egger’s test: P=0.068), proving that supplementation with *S. boulardii* can improve the eradication rate of the standard protocol.

#### Layered analysis

Subgroup analysis stratified by patient age indicated that the eradication rate in children (RR=1.14, 95% CI: 1.05–1.25, P=0.002) was 5% higher than that in adults (RR=1.09, 95% CI: 1.04–1.14, P=0.0002).

Subgroup analysis stratified by study location revealed that the eradication rate in China (RR=1.12, 95% CI: 1.04–1.19, P=0.001) was 2% higher than that in foreign countries (RR=1.10, 95% CI: 1.05–1.15, P=0.0001).

Subgroup analysis stratified by the standard eradication protocol showed that the eradication rate with quadruple therapy (RR=1.12, 95% CI: 1.06–1.19, P=0.0002) increased by 7% when compared with triple therapy (RR=1.05, 95% CI: 1.00–1.11, P=0.04). Moreover, the eradication rate of sequential therapy increased by 3% when compared with quadruple therapy.

Subgroup analysis stratified by medication duration showed that there was no significant difference between the 7-day therapy (RR=1.09, 95% CI: 1.03–1.17, P=0.006) and the 14-day therapy (RR=1.10, 95% CI: 1.04–1.16, P=0.0007), with only a 1% difference. However, the addition of *S. boulardii* to the 7-day therapy did not increase the eradication rate at 4 weeks (**Table 2**).

**Table 2:** Layered analysis of eradication rate.

Subgroup	Number of Articles	Sample size	RR(95%)	P	I^2^(%)
Stratified by age					
Children	4	492	1.14 (1.05,1.25)	0.002	0
Adults	14	3215	1.09 (1.04,1.14)	0.0002	37
Stratified by study location					
China	9	2096	1.12 (1.04,1.19)	0.001	51
Foreign	9	1611	1.10 (1.05,1.15)	0.0001	0
Stratified according to the standard eradication protocol					
Triple therapy	5	1232	1.05 (1.00,1.11)	0.04	0
Quadruple therapy	12	2276	1.12 (1.06,1.19)	0.0002	42
Sequential treatment	1	199	1.15 (1.00,1.32)	0.05	
Stratified by medication course					
7-day therapy	5	970	1.09 (1.03,1.17)	0.006	0
14-day therapy	13	2538	1.10 (1.04,1.16)	0.0007	44

Subgroup analysis of the eradication rate of *S. boulardii* as an adjuvant therapy for *H. pylori*. RR, relative risk; P, probability.

### Secondary outcome: adverse effects

#### Total adverse effects

**Figure 4:**
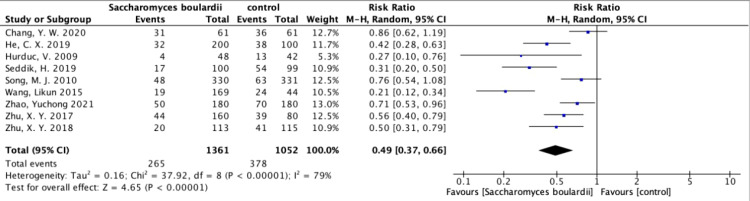
Forest plot analyzing the total adverse effects of *S. boulardii* as an adjuvant therapy for *H. pylori*.

Among the 19 included articles, 9 analyzed the differences in overall adverse effects after the addition of *S. boulardii* (**Figure 4**), and these studies showed high heterogeneity (I^2^ = 79%, P<0.00001). In total, the overall adverse effects decreased by 51% (RR=0.49, 95% CI: 0.37–0.66, P<0.00001) when using random effects combined with effect measures.

### Other adverse effects

#### Diarrhea

We performed a meta-analysis on 16 articles reporting the incidence of diarrhea. These studies had moderate heterogeneity (I^2^ = 58%, P=0.002). Using random effects combined with effect measures, we found that the incidence of diarrhea decreased by 64% (RR=0.36, 95% CI: 0.26–0.48, P<0.0001) with *S. boulardii* supplementation.

#### Abdominal pain

A meta-analysis was conducted on nine articles reporting the incidence of abdominal pain, and we found that these studies had no heterogeneity (I^2^ = 0%, P=0.79). Using a fixed-effects model, we found no significant difference in the incidence of abdominal pain between the *S. boulardii* and control groups (RR=0.78, P=0.10). Therefore, the addition of *S. boulardii* did not reduce the incidence of abdominal pain.

#### Abdominal distension

We conducted a meta-analysis on seven articles with mild heterogeneity (I^2^ = 43%, P=0.10) reporting the incidence of abdominal distension. Random effects were used to merge the effects, and we found that the abdominal distension rate decreased by 51% (RR=0.49, 95% CI: 0.33–0.72, P=0.0004) with *S. boulardii* supplementation, without bias (Egger’s test: P=0.994).

#### Constipation

A meta-analysis was conducted on six articles reporting the incidence of constipation. These studies had no heterogeneity (I^2^ = 0%, P=0.48). Therefore, we used a fixed-effects model and found that the addition of *S. boulardii* significantly reduced the incidence of constipation (RR=0.38, 95% CI: 0.26–0.57, P<0.00001). The results were unbiased (Egger’s test: P=0.973).

#### Nausea

We performed a meta-analysis on 12 articles with mild heterogeneity (I^2^ = 48%, P=0.03) reporting the incidence of nausea. Random effects were used to merge the effects, and we found that the nausea rate decreased by 50% (RR=0.50, 95%: 0.37–0.68, P<0.00001) with *S. boulardii* supplementation. The results were statistically significant and unbiased (Egger’s test: P=0.095).

#### Vomiting

A meta-analysis was conducted on eight articles reporting the incidence of vomiting. These articles had mild heterogeneity (I^2^ = 38%, P=0.12). Random effects were used to merge the effects, and we found that the results were not statistically significant (RR=0.71, P=0.07). Therefore, the addition of *S. boulardii* did not reduce the incidence of vomiting.

#### Taste disorders

We conducted a meta-analysis on seven articles that studied the incidence of taste disorders and found moderate heterogeneity (I^2^ = 64%, P=0.01). Random effects were used to merge the effects, and the results were not statistically significant (RR=0.82, P=0.4). Therefore, the addition of *S. boulardii* did not reduce the incidence of taste disorders (**Table 3**).

**Table 3:** Incidence rate of other adverse reactions.

Adverse reactions	Number of Articles	Sample size	RR (95%)	P	I^2^(%)
diarrhea	16	3701	0.36 (0.26-0.48)	<0.00001	58
abdominal pain	9	2153	0.78 (0.57-1.05)	0.1	0
abdominal distension	7	1308	0.49 (0.33-0.72)	0.0004	43
constipation	6	1324	0.38 (0.26-0.57)	<0.00001	0
nausea	12	2505	0.50 (0.37-0.68)	<0.00001	48
vomit	8	1744	0.71 (0.50-1.02)	0.07	38
taste disorders	7	1417	0.82 (0.51-1.31)	0.4	64

Effect of *S. boulardii* as an adjuvant therapy for *H. pylori* on adverse effects. RR, relative risk; P, probability.

## Discussion

In this meta-analysis, we investigated changes in the rates of *H. pylori* eradication after the addition of *S. boulardii* to eradication therapy using ITT analysis. In the ITT analysis, we found that the addition of *S. boulardii* increased the eradication rate by 11%, with statistically significant and non-heterogeneous results consistent with those of a previous meta-analysis published by Zhou et al. in 2019 (Zhou et al., 2019). In the two latest RCTs published in 2022—one of which was a triple-protocol study by Yang et al (Yang et al., 2022).including 497 patients—the eradication rate increased by 12.6% after the addition of *S. boulardii*, which is consistent with our results. Another RCT performed by Naghibzadeh et al. (2022) found that the addition of *S. boulardii* increased the eradication rate by 7.7% in 156 patients undergoing quadruple therapy; however, this increase was not statistically significant. This might have been due to various factors such as the inclusion of a placebo in the experiment, the fact that quadruple therapy itself improved the eradication effect compared to triple therapy, or an insufficient dosage of *S. boulardii*.

When we studied the total adverse effects reported by the included studies, we found a high degree of heterogeneity in their results. Further investigation of L’Abbé and radial plots confirmed this high degree of heterogeneity. Upon conducting a sensitivity analysis of these studies, we found that deleting any articles did not cause a significant change in the results. Additionally, the sensitivity analysis did not identify the source of heterogeneity. Therefore, we conducted a subgroup analysis from multiple dimensions; however, we did not find any sources of heterogeneity, indicating a reliable 51% reduction in total adverse effects. This conclusion is consistent with the results of a previous meta-analysis by Zhou et al (Zhou et al., 2019), showing that the addition of *S. boulardii* can reduce the total adverse effects of eradication therapy.

When studying the incidence of diarrhea, we examined the L’Abbé and radial plots again and found moderate heterogeneity. A sensitivity analysis was conducted on these studies, and it was found that the study by Seddik et al. (2019) had a significant impact on heterogeneity. After excluding this study, the I^2^ decreased to <50%. This study was the only treatment that used sequential therapy; therefore, we concluded that this study might have been a source of heterogeneity. Owing to antibiotic resistance and the adverse effects of bismuth, sequential therapy can serve as a replacement for traditional triple or quadruple therapy failures (Yang et al., 2014). A study by Zullo et al. (2003) including 1,749 patients showed that sequential therapy had a higher eradication rate than traditional therapy (92% vs. 74%). Therefore, sequential therapy may become a new-generation first-line treatment option in the future; however, further experimental studies are required to confirm its efficacy and safety.

In this meta-analysis, we concluded that the addition of *S. boulardii* to eradication therapy significantly reduced the incidence of abdominal distension, constipation, and nausea, with no heterogeneity or only mild heterogeneity. Similarly, a recent triple-protocol RCT in China (Yang et al., 2022) showed a significant improvement in abdominal distension with *S. boulardii* supplementation. Our findings are also consistent with the results of a previous meta-analysis by Zhou et al. (2019) that investigated the incidence of diarrhea, bloating, constipation, and nausea with eradication therapy. These results confirm that the addition of *S. boulardii* can reduce the occurrence of these adverse reactions, providing a theoretical basis for clinical practice.

After conducting a stratified analysis of 18 articles, we found that the eradication rate in the child group increased by 5% when compared with that in the adult group. This is consistent with Zhou et al.’s (Zhou et al., 2019) finding that the eradication rate in children increased by 3.25% when compared with that in adults. Additionally, we found that the eradication rate of quadruple therapy was higher than that of triple therapy, with an increase of 7%. However, sequential therapy had the highest eradication rate, with an increase of 9% when compared with quadruple therapy. Notably, sequential therapy has several advantages over traditional therapy, with some studies reporting that sequential therapy can increase the eradication rate by 18% when compared with traditional therapy (Zullo et al., 2003). This presents new possibilities for clinical treatment. In this study, we also compared the eradication rates at different study locations and medication durations; however, we found no significant differences in these factors.

The present meta-analysis evaluated the incidence of other adverse effects such as abdominal pain, vomiting, and taste disorders. Although the incidence of these adverse effects decreased with the addition of *S. boulardii*, these results were not statistically significant. Consistent with our results, Naghibzadeh et al. (2022) found no significant improvement in the incidence of vomiting and taste disorders with the addition of *S. boulardii*. Since there is currently limited clinical research regarding whether the addition of *S. boulardii* can decrease these adverse effects, these results may still hold some significance for clinical practice. However, further studies are required to confirm this hypothesis.

Previous studies have also shown that other probiotics may increase the eradication rate of *H. pylori* and reduce adverse effects. For example, Naghibzadeh et al. (2022) studied a group of treatment regimens using *L. reuteri* in combination with quadruple therapy and found a small improvement in the eradication rate; however, these results were not statistically significant. Similarly, Zhu et al. (2018) studied a treatment regimen using *L. reuteri* and found a 2.5% increase in the eradication rate, which was also not statistically significant. Previous research has shown that the colonization of lactobacilli in the stomach can reduce the production of gastritis, promote mucus regeneration, downregulate the expression of cag pathogenicity island genes (García et al., 2012), and prevent the colonization of *H. pylori* through specific adhesives. Another prospective trial statistically analyzed the efficacy of combining four probiotics (*Lactobacillus acidophilus*, *Lactiplantacillus plantarum*, *Bifidobacterium lactis*, and *S. boulardii*) and found that combining multiple probiotics can improve eradication rates and reduce the incidence of adverse effects (Viazis et al., 2022). However, further randomized trials are required to confirm the efficacy of these probiotics.

Previous researchers have also studied patients’ compliance with the addition of *S. boulardii* to *H. pylori* eradication therapy. Seddik et al. (2019) found that adding *S. boulardii* to the sequential treatment increased treatment compliance by 3.8% (95.0% vs. 91.2%, P<0.001). Similarly, in an RCT involving children, Bin et al. (2015) found that no patient in the treatment group terminated *H. pylori* treatment prematurely, whereas six patients in the control group stopped treatment without medical advice, with a statistically significant difference (p=0.027). However, due to the small sample size of their study, further investigation is required to determine whether *S. boulardii* can improve patient compliance with eradication therapy.

## Conclusion

We found that adding *S. boulardii* to traditional eradication treatment methods can improve the *H. pylori* eradication rate and reduce the total adverse effects and incidence of diarrhea, bloating, constipation, and nausea. However, it did not reduce the incidence of abdominal pain, vomiting, and taste disorders.

## Data Availability

The original contributions presented in the study are included in the article/supplementary material. Further inquiries can be directed to the corresponding author/s.
